# The heart-mind relationship in women cardiovascular primary prevention: the role of depression, anxiety, distress and Type-D personality in the 10-years cardiovascular risk evaluation

**DOI:** 10.3389/fcvm.2024.1308337

**Published:** 2024-03-07

**Authors:** Mattia Giuliani, Giulia Santagostino Baldi, Nicolò Capra, Alice Bonomi, Chiara Marzorati, Valeria Sebri, Paolo Guiddi, Piero Montorsi, Gabriella Pravettoni, Daniela Trabattoni

**Affiliations:** ^1^ Psychology Division, Centro Cardiologico Monzino, IRCCS, Milan, Italy; ^2^ Department of Interventional Cardiology and Women Heart Center, Centro Cardiologico Monzino, IRCCS, Milan, Italy; ^3^ Biostatistic Unit, Centro Cardiologico Monzino, IRCCS, Milan, Italy; ^4^ Applied Research Division for Cognitive and Psychological Science, Istituto Europeo di Oncologia (IEO), European Institute of Oncology IRCCS, Milan, Italy; ^5^ Department of Clinical Sciences and Community Health, University of Milan, Milan, Italy; ^6^ Department of Oncology and Hemato-Oncology, University of Milan, Milan, Italy

**Keywords:** anxiety, depression, distress, Type-D personality, gender medicine, cardiovascular prevention

## Abstract

**Introduction:**

Cardiovascular diseases are the leading cause of death among women. Prevention programmes underscore the need to address women-specific risk factors. Additionally, mental well-being is a significant aspect to consider when grappling with cardiovascular disease in women, particularly depression, anxiety, distress, and personality traits. This study aimed to create “at-risk” psychological profiles for women without prior cardiovascular disease history and to evaluate the association between anxiety, depression, distress, and Type-D personality traits with increased cardiovascular risk over 10 years.

**Methods:**

219 women voluntarily participated in the “Monzino Women's Heart Centre” project for primary prevention and early diagnosis of cardiovascular diseases. Psychological profiles were developed utilising cluster analysis.

**Results:**

The primary finding indicating that belonging to the “at-risk” psychological cluster was associated with a surge in the 10-year cardiovascular risk prediction score, despite the number of comorbid risk factors (Psychological “at-risk” cluster: *β* = .0674; *p* = .006; Risk factors: *β* = .0199; *p* = .242).

**Conclusions:**

This finding suggests that psychological well-being of women should be assessed from the very beginning of cardiovascular prevention programmes.

## 1 Introduction

Cardiovascular diseases (CVD) is the primary cause of death for women, responsible for 35% of global mortality in 2019 (1). International calls for sex-specific cardiovascular research have been made due to disparities in CVD treatment and prognostic outcomes between sexes (1). In recent years, there has been a renewed impetus for investigating CVD in women and evidence on female-specific CVD risk factors has increased (2). A broader perspective is essential when it comes to women's health. Clinicians and researchers should consider psycho-social, economic, and cultural factors that contribute to the development, progression, and exacerbation of CVD. Unfortunately, such factors are still mostly ignored. In addition to the well-established CVD risk factors (hypertension, dyslipidemia, diabetes, obesity, unhealthy diet, sedentary lifestyle, and smoking), women also present sex-specific risk factors. These include menopause and premature menopause, pregnancy-related complications (gestational diabetes, hypertension and dyslipidemia during pregnancy, pre-eclampsia and preterm delivery), polycystic ovary syndrome, and systemic and autoimmune disorders (1, 2). Nonetheless, psychological symptoms and disorders, including depression, anxiety and distress, which are acknowledged risk factors for CVD (3–9), are more often detected in women than men (10, 11). According to the narrative review by Vlachopoulou et al. (12), depression in women is strongly linked to CVD. Depression is known to be a risk factor for fatal coronary heart disease (CHD) in women who have no baseline CHD, and a high risk for CHD in women with both Type 1 and Type 2 diabetes. Additionally, depression is independently predictive of coronary artery disease (CAD) in women, along with hypertension, waist-hip ratio and physical inactivity (12). The authors emphasized the role of anxiety, observing that women with high levels of phobic anxiety face a greater risk of fatal CHD, and that CVD events can be predicted in women with suspected myocardial ischemia who exhibit comorbid depression and anxiety symptoms (12). There is a wide and compelling evidence linking psychological distress with earlier onset of CVD, faster disease progression, worse prognosis, and an increased risk of mortality (13–17). Nonetheless, psychological distress is associated with stress-induced cardiomyopathy, which is also known as Takotsubo or “Broken Heart Syndrome”. This condition mainly affects postmenopausal women and is typically triggered by extreme physical or emotional events (18–20). In a recent study, Pimple et al. demonstrated that high psychological distress levels were linked to future cardiovascular events among female patients diagnosed with CAD (21). Psychological distress is the primary characteristic of “Type-D personality”, characterized by persistent negative emotions, such as anger, contempt, disgust, fear, guilt, nervousness, and low self-esteem (i.e., negative affectivity), alongside with social inhibition, a stable tendency to inhibit the expression of emotions and behaviours in social interaction (22). Type-D personality has a significant impact on various outcomes in cardiac patients. It is linked to poorer prognosis in patients with CAD—specifically, twice the risk of mortality and nonfatal myocardial infarction (MI). It is also a predictor of worse health status in CAD and heart failure (HF) patients, and is associated with worse quality of life in CAD patients undergoing rehabilitation. Type-D personality is linked to poor adherence to treatment and physicians’ prescriptions, and higher serum inflammatory marker (23). Based on the available evidence, it is crucial to identify and address women's specific psychological and cardiovascular risk factors in a timely manner for the improvement of their cardiovascular health (1). The aim of this study was to test the hypothesis that women without cardiovascular disease, but with a psychological profile deemed at risk –due to the presence of depressive, anxiety and distress symptoms and Type-D personality- may be more susceptible to a cardiovascular event within 10 years than those free from such psychological features. To our knowledge, this is the first study exploring such an association within healthy females.

## 2 Methods

### 2.1 Sample

219 women volunteered to participate in the “Monzino Women's Heart Centre” project for primary prevention and early diagnosis of cardiovascular disease, as described in Gili et al. (24). The participants were consecutively included in the sample and had to meet two criteria: they had to be women aged between 35 and 65 years, and had no history of overt or previous CVD. Furthermore, all participants provided a written informed consent, which was approved by the Centro Cardiologico Monzino Ethical Committee.

### 2.2 Psychological questionnaires

All the following questionnaires were used in their Italian validated versions (25–28).

#### 2.2.1 Beck depression inventory—II (BDI-II)

The BDI-II is a self-report questionnaire containing 21 items that evaluate the severity of depressive symptoms in adults and adolescents (29). The patient rates each item on a Likert scale of 0–3. Then, the total score is calculated by summing the scores from all the items, which will determine the severity of the depressive symptoms into four levels: minimal depressive symptoms (from 0 to 13), mild depressive symptoms (from 14 to 19), moderate depressive symptoms (from 20 to 28) and severe depressive symptoms (from 29 to 63). The BDI-II has exhibited a high level of internal consistency (Cronbach *α* = .80), good convergent validity when compared with the Depression Questionnaire, and good test-retest reliability after one month (*r* = .76) (28).

#### 2.2.2 State-trait anxiety inventory—Y form (STAI-Y)

The STAI-Y is a frequently used self-report inventory that evaluates both state and trait anxiety (30). The questionnaire contains 20 items that measure the level of state anxiety (STAI-S). State anxiety refers to anxiety experienced at the time of the evaluation, which is considered context-dependent and transient. Additionally, the STAI-Y comprises 20 further items that measure the levels of trait anxiety (STAI-T). Trait anxiety is usually experienced in day-to-day life and is thought to be a stable, context-independent condition. All items are rated on a 4-point scale with a total score ranging from 20 to 80 for both the state and trait anxiety subscales. The STAI-Y demonstrated a high level of internal consistency, with a Cronbach α of.93 for state anxiety and .90 for trait anxiety (31). Only the STAI-T scale was employed in this study.

#### 2.2.3 Perceived stress scale (PSS)

The PSS is the most commonly used self-report measure for evaluating individuals’ distress perception (32). The questionnaire examines the frequency of feelings and thoughts related to distress perception over the past month. The responses range from 0 to 40, with higher scores corresponding to a greater level of perceived distress. Interpretation of the scores is based on three value categories: low distress perception (from 0 to 13); moderate distress perception (from 14 to 26); and high distress perception (from 27 to 40).

#### 2.2.4 Distressed personality scale (DS-14)

The DS-14 is a concise self-report survey that has been utilized globally for assessing negative affectivity (NA) and social inhibition (SI), associated with Type-D personality (22). The DS-14 comprises 14 items, each rated on a scale ranging from 0 (i.e., false) to 4 (i.e., true). This questionnaire provides distinct scales for both NA and SI, with scores ranging from 0 to 28. An individual with a score of ≥10 in the NA and/or SI scale is more likely to exhibit the correspondent personality trait.

### 2.3 Medical data

Baseline clinical data were collected for all patients, encompassing biometric data, essential parameters including arterial blood pressure, conventional cardiovascular risk factors (i.e., smoking habit, familial history of cardiovascular disease, hypertension, dyslipidemia, diabetes mellitus, obesity/overweight), and cardiovascular risk factors specific to women. The participants’ medical records were thoroughly examined, including their past medical history, with a specific focus on endocrine, autoimmune, and gynecological disorders, as well as any instances of early or premature menopause, gestational diabetes and/or hypertension, eclampsia, or pre-term delivery. Each participant was provided with a conclusive summary detailing their estimated risk of cardiovascular event, calculated using the Progetto Cuore score: 10-year CV Risk PC (https://www.cuore.iss.it/valutazione/calc-rischio).

### 2.4 Statistical analyses

A k-means cluster analysis was conducted to identify subject subgroups with shared psychological characteristics, therefore using as clusterization variables the standardized scores of BDI-II, STAI-T, PSS, and both DS-14 NA and SI. The k-means algorithm was repeated 100 times with random initial centroids to ensure repeatability and stability of results, and the best configuration was selected. A scree plot was used to assess the Within-Cluster-Sum of Squares (WSS) trend based on the number of clusters, while the elbow method identified the optimal number. Mean ± standard deviation or median and [interquartile range] were used to express continuous variables, while categorical variables were presented as absolute numbers and percentages. Unpaired t-tests were used to compare clusters for normally distributed variables, while Wilcoxon rank sum tests were used for skewed variables. Categorical variables were analyzed using either Chi-square test or Fisher's exact test. To assess trends across clusters for categorical variables, the Mantel-Haenszel *χ*^2^-test was employed. Non-normally distributed variables were log-transformed. General linear models were used to investigate the association between estimated cardiovascular event risk, cluster membership, and number of conventional and female-specific CVD risk factors. Missing data were imputed using the PROC MI (Multiple imputation) procedure via the MCMC (Markov Chain Monte Carlo) method. One dataset was randomly selected out of the 25 imputed ones for the main analysis. To assess consistency, we calculated the percentage of times that the *p*-value was found to be significant or non-significant. We utilized the k-means package of RStudio (V. 4.0.3, RStudio, Boston, MA, USA) to perform k-means cluster analysis, while all other statistical analyses were conducted using SAS software package (V. 9.4, SAS Institute Inc., Cary, NC). All tests were two-sided, with a significance level of *p* ≤ .05.

## 3 Results

### 3.1 Cluster analysis

**Figure 1:**
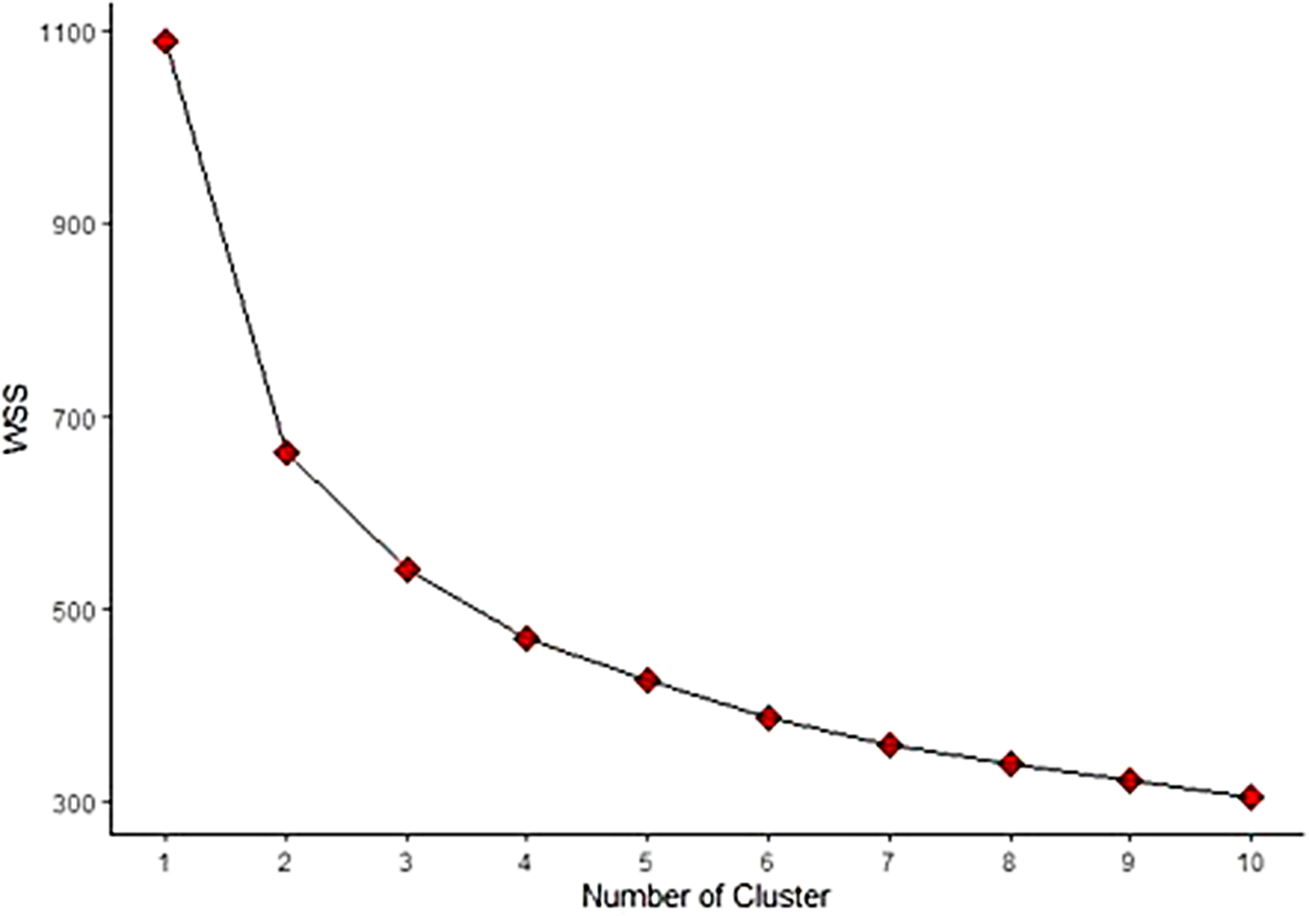
Scree plot for k-means clustering of psychological data. The scree plot shows how Within-Cluster-Sum of Squares (WSS) changes as a function of the number of clusters identified using the k-means algorithm. The elbow method identified an optimal number of clusters equal to two, the point at which the graph shows a distinct change in slope.

Cluster analysis was utilized to categorize the 219 subjects into groups that share similar psychological characteristics. The elbow method applied to the scree plot (Figure 1) identified two as the most suitable number of clusters. As a result, the participants were divided into two subgroups: Cluster 1 or NPR (No Psychological Risk) cluster, consisting of 132 individuals, and Cluster 2 or PR (Psychological Risk) cluster, consisting of 87 individuals.

#### 3.1.1 NPR and PR clusters sociodemographic features

Table 1 displays the baseline characteristics of our population. Overall, both NPR and PR cluster participants were middle-aged and highly educated -with the majority having completed high school or attained a graduate degree- and a high proportion being married. No significant differences were observed between the two clusters.

**Table 1:** NPR and PR clusters sociodemographic features.

		NPR (*N* = 132)		PR (*N* = 87)		*p*-value
		*Mean*	*SD*	*Mean*	*SD*	
Age (years)		52.18	6.07	53.62	6.71	.101
		*Frequency*	*%*	*Frequency*	*%*	** **
Education	*Elementary school*	0	0	0	0	.611
	*Intermediate school*	4	3.08	5	5.75	
	*High school*	62	47.69	44	50.57	
	*University*	56	43.08	35	40.23	
	*Post-university (i.e., PhD)*	8	6.15	3	3.45	
Marital status	*Single*	15	11.54	14	16.28	.695
	*In a relationship*	8	6.15	7	8.14	
	*Married*	91	70	58	67.44	
	*Divorced*	15	11.54	7	8.14	
	*Widow*	1	0.77	0	0	
Classic risk factors		*Frequency*	*%*	*Frequency*	*%*	** **
Smoking habit	*Smoker*	100	75.76	55	63.22	.**046**
	*Non-smoker*	32	24.24	32	36.78	
Physical activity	*Sedentary*	53	40.15	43	49.43	.176
	*Occasional/Regular*	79	59.85	44	50.57	
Hypertension	*Present*	56	42.42	40	45.98	.604
	*Absent*	76	57.58	47	54.02	
Diabetes	*Present*	17	12.88	11	12.64	.959
	*Absent*	115	87.12	76	87.36	
Obesity	*Present*	11	8.33	9	10.34	.613
	*Absent*	121	91.67	78	89.66	
Dyslipidaemia	*Present*	22	16.67	14	16.09	.911
	*Absent*	110	83.33	73	83.91	
Women Specific risk factors		*Frequency*	*%*	*Frequency*	*%*	** **
Polycystic ovary syndrome	*Present*	6	4.55	5	5.75	.757
	*Absent*	126	95.45	82	94.25	
Preterm delivery	*Present*	4	3.03	1	1.15	.650
	*Absent*	128	96.97	86	98.85	
Eclampsia	*Present*	4	3.03	0	0	.154
	*Absent*	128	96.97	87	100	
Gestational hypertension	*Present*	5	3.79	3	3.45	1.000
	*Absent*	127	96.21	84	96.55	
Gestational diabetes	*Present*	3	2.27	0	0	.278
	*Absent*	129	97.73	87	100	
* *	* *	*Median*	*IQR*	*Median*	*IQR*	
10-years CV Risk PC		1	0.4;1.4	1.2	0.8;1.9	.**007**

Bold values indicate significant *p*-values <.05.

#### 3.1.2 Classic and women specific risk factors prevalence in NPR and PR clusters

Cigarette smoking was the most common modifiable traditional risk factor in both clusters, and the only one that showed a significant difference between them -NPR cluster displayed a higher percentage. Following this were hypertension and sedentary lifestyle, which were prevalent in both clusters. As for women-specific risk factors, polycystic ovary syndrome was the most common, but there was no significant difference between the two clusters. The 10-year CV Risk PC was generally low, but the PR cluster had a significantly higher score. These data are shown in Table 1.

**Figure 2:**
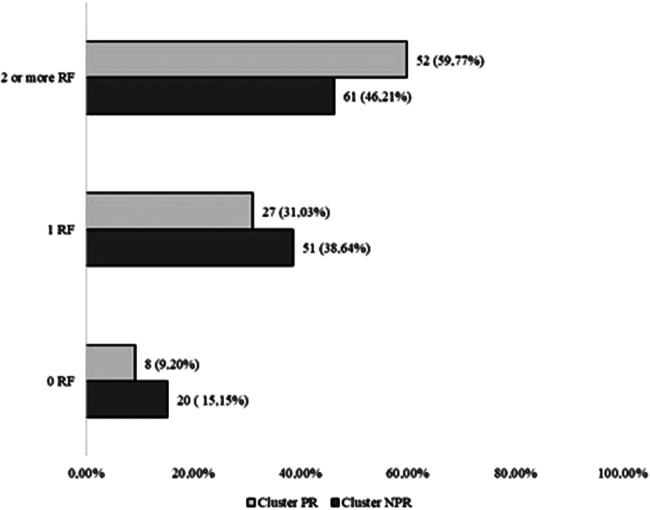
Classic and women specific RF trends in PR and NPR clusters.

The classic and gender-specific risk factors’ frequency increased significantly (*p*-trend = .04) in the PR compared to the NPR group (Figure 2). This result received validation in 24 out of 25 imputations (96%).

#### 3.1.3 NPR and PR clusters psychological features

The NPR cluster displayed low levels of anxiety symptoms, minimal depressive symptoms, and a low distress score. In contrast, the PR cluster demonstrated high levels of anxiety symptoms, mild depressive symptoms, and a moderate distress score. Additionally, the NPR cluster exhibited neither negative affectivity nor social inhibition, while the PR cluster showed clinically relevant levels of both personality traits. All these results are reported in Table 2.

**Table 2:** NPR and PR clusters psychological features.

	NPR (*N* = 132)		PR (*N* = 87)		*p*-value
	Mean/ Median	SD/ IQR	Mean/ Median	SD/ IQR	
BDI-II	6	3;8	16	13;20	**<**.**0001**
STAI-T	37.98	6.95	49.59	6.13	**<**.**0001**
PSS	13.54	5.07	21.85	4.7	**<**.**0001**
DS-14 (NA)	7.42	4.35	17.18	5.36	**<**.**0001**
DS-14 (SI)	5.5	2.5;9	10	6;13	**<**.**0001**

BDI-II, beck depression inventory-II; STAI-T, state-trait anxiety inventory (trait anxiety); PSS, perceived stress scale; DS-14 (NA), distress personality—14, negative affectivity scale; DS-14 (SI), distress personality—14, social inhibition scale.

Bold values indicate significant *p*-values <.05.

### 3.2 Multivariate regression analysis on CV Risk PC

The principal finding of this analysis indicates that membership in the PR cluster characterized by an elevated psychological risk is linked to an elevation in 10-years CV Risk PC (expressed in logarithmic scale). This is regardless of the number of risk factors (both classic and women-specific) concurrently presented by subjects in comorbidity (Table 3). Our results were substantiated in all 25 imputations (100%).

**Table 3:** Multivariate regression analysis on 10-years CV risk PC.

	β	95% CL		*p*-value
PR cluster	.0674	.0193	.1155	**.** **006**
Risk factors (both classic and women specific)	.0199	−.0135	.0534	.242

Bold values indicate significant *p*-values <.05.

## 4 Discussion

This study was the first to examine the association between a high 10-years CV risk and a psychological profile at risk, including the presence of anxiety, depressive and distress symptoms, and Type-D personality traits, within a cohort of healthy female participants. The primary finding showed that women with a psychological profile at risk scored higher in 10-years CV risk measure, irrespective of the number of comorbid risk factors. This evidence is of significance in the field of cardiovascular prevention for women as it indicates that addressing traditional psychological risk factors (such as anxiety, depression, distress and Type-D personality traits) for CVD is crucial from the earliest stage of prevention for women.

### 4.1 Theoretical background

The study's theoretical background is based on two main models: Gordon's Operational Model (33) and Felner and Silverman's Antecedent Condition Model (34). We adopted the concept of “indicated interventions” from the Operational Model, which refers to screening programmes for specific subgroups of the population who present one or more specific risk conditions for the development of a disease. In this case, the subgroup consists of women who do not have an overt heart disease but may have sex-related risk factors for its development. The Antecedent Condition Model distinguishes between predisposing and precipitating factors for a specific clinical condition. In this case, sex and sex-specific factors for cardiovascular disease represent the predisposing factors, while the concomitant presence of cardiovascular risk factors and unfavourable psychological characteristics represent the precipitating factors. Both models recommend targeted interventions from the earliest stages of prevention to reduce diseases risk exposure.

### 4.2 Sociodemographic and risk factors

Both clusters show no statistically significant difference in demographic composition. They comprise of married middle-aged women with medium to high levels of education, i.e., high school and university. Furthermore, there are no discernible differences in the frequency of classic, female-specific risk factors, except for smoking. The NPR cluster has a higher frequency of smokers than the PR cluster. However, it should be acknowledged that the borderline significance (*p*-value = .046) may be attributed to the smaller sample size of the PR cluster. We argue that it is highly probable that this significance may disappear with an increase in the sample size. Therefore, the two clusters can be considered similar in terms of demographic composition and risk factors. This information is crucial for interpreting the significant result regarding the effect of belonging to the psychological risk cluster on 10-year CV risk. Therefore, the primary interest lies in the role of psychological risk factors in increasing the 10-year risk of a cardiovascular event. In fact, anxiety, depression, distress, and Type-D personality were the only significant differences between the two clusters.

### 4.3 Depressive, anxiety, distress, type-D personality and 10-years CVD risk in women

To the best of our knowledge, only one study has examined the relationship between depression in women and a widely-accepted lifetime CVD risk measure: the 10-year atherosclerotic CVD (ASCVD) (35). The researchers reported that, in women aged 40–79, the absolute risk of ASCVD was 6.0% for those without depression, 6.9% for those with mild depression, and 7.6% for those with major depression. They also found that among women aged 20–39, the prevalence of high lifetime CVD risk was 41.9% for those whitout depression, 53.2% for those with mild depression, and 66.5% for those with major depression. Our own findings align with these results, indicating an increasing trend in ASCVD risk with severity of depressive symptoms. The relationship between depression/depressive symptoms and CVD is a well-established issue. In 2008, the AHA consensus report in which it was clearly outlined that depression acts as an independent risk factor for CVD, and especially for CAD and MI (36). Nonetheless, to date, only a limited number of specialized hospitals have incorporated depression screening as part of their daily procedures (9). Currently, there is a lack of studies examining the relationship between anxiety and distress symptoms and the 10-year CVD risk in women. Consequently, we must refer to research investigating the relationship between anxiety, distress and overall cardiovascular risk to discuss our findings. According to Mosarla and Wood, anxiety symptoms heighten the risk of CVD in females, leading to a lower quality of life, and an increased cardiovascular morbidity, and mortality (37). Matthews et al. (38) conducted a study on a cohort of healthy postmenopausal women, which demonstrated that those who with higher levels of anger, hostility, and anxiety exhibited greater carotid thickening as assessed by IMT, even after a one-year follow-up period. In another prospective cohort study, researchers found that a one standard deviation increase in psychological distress was associated with a 44% elevated risk of cardiovascular events solely in women with CAD (21). Type-D personality traits, specifically NA and SI, are strongly correlated with both depression and anxiety (39). It is plausible to that Type-D personality traits may increase susceptibility to the development or worsening of anxiety and depressive symptoms, thereby contributing to an increased the risk of cardiovascular events. A fascinating investigation by Al-Qezweny et al. indicated that patients who underwent a percutaneous coronary intervention with type D personality had a 3.69-fold heightened risk of depression and a 2.72-fold heightened risk of anxiety at 10year of follow-up (40). The present study aligns with previous evidence. Anxiety symptoms and distress, akin to depressive symptoms, ought to receive appropriate screening and treatment from the early stages of cardiovascular prevention in women.

### 4.4 Limitations

This paper has certain limitations; thus, the conclusions need to be interpreted with following elements in mind. First and foremost, the study exclusively involves Caucasians, and previous research indicates that cardiovascular risk is influenced by the participant's ethnic background (41). Secondly, the algorithm for calculating 10-year CVD risk requires consideration. The study utilized the 10-year CV risk PC algorithm, but it is crucial to examine whether the outcomes remain consistent with other algorithms employed in literature. Thirdly, one must also consider the psychological survey instruments employed in the present study. Validated instruments that are extensively used in literature to assess anxiety, depressive and distress symptoms, and Type-D personality traits were utilized. Nevertheless, these questionnaires are subject to limitations typical of all self-report measures and thus require further validation with other questionnaires that examined the same psychopathological constructs. A final point to consider is that the study sample consisted only of who were aware of the project and/or knew about the hospital, potentially introducing a sampling bias.

## 5 Conclusions and future directions

Gender disparity is a recognized concern in cardiovascular research, as women are frequently underrepresented in study samples and their specific characteristics, needs, and treatment within the cardiovascular field remain inadequately understood. This original research focuses on evaluating the potential impact of psychological well-being on the risk of developing cardiovascular disease in women who have no prior history of such conditions, over a ten-year period. Surprisingly, the findings indicate that psychological well-being is a stronger predictor than traditional cardiac risk factors (such as hypertension, diabetes, and smoking) for the development of cardiovascular disease within a decade, among women with no prior history of such disease. Although these results should be approached with caution, they lead to two primary considerations: (1) it is essential to evaluate the psychological well-being of patients from the earliest prevention stages; (2) while the link between anxiety, depression, perceived distress, personality traits and cardiovascular risk has been widely acknowledged in literature, this study confirms its strong association. To further investigate the focus of this research, future prospects will be considered: (1) to increase the sample size; (2) to include a cohort of men without any cardiovascular pathology to evaluate potential differences in the incidence of certain psychopathological traits and the role of psychological well-being on cardiovascular risk; (3) to include participants of different ethnicities to ensure a diverse sample; and (4) to collaborate with other hospitals that offer comparable services to “Monzino Women” to minimize sampling bias.

## Data Availability

The datasets presented in this study can be found in online repositories. The names of the repository/repositories and accession number(s) can be found below: 10.5281/zenodo.5807307.
