# Supplementary material for: The heart-mind relationship in women cardiovascular primary prevention: the role of depression, anxiety, distress and Type-D personality in the 10-years cardiovascular risk evaluation
Source: Front Cardiovasc Med. 2024 Mar 7;11:1308337. doi: 10.3389/fcvm.2024.1308337 (PMC10955135; doi:10.3389/fcvm.2024.1308337)
Supplement: Supplementary file 1 [file Table1.docx]

**Supplementary Table 1.** Classic and Women specific risk factors prevalence in NPR and PR clusters in each imputation

|  |  | NPR | PR | p-value |
| --- | --- | --- | --- | --- |
| Imputation 1 | 2 or more RF | 61 (46,21%) | 52 (59,77%) | **0,039** |
|  | 1 RF | 50 (37,88%) | 27 (31,03%) |  |
|  | 0 RF | 21 (15,91%) | 8 (9,20%) |  |
| Imputation 2 | 2 or more RF | 61 (46,21%) | 52 (59,77%) | **0,044** |
|  | 1 RF | 51 (38,64%) | 27 (31,03%) |  |
|  | 0 RF | 20 (15,15%) | 8 (9,20%) |  |
| Imputation 3 | 2 or more RF | 60 (45,45%) | 52 (59,77%) | **0,032** |
|  | 1 RF | 51 (38,64%) | 27 (31,03%) |  |
|  | 0 RF | 21 (15,91%) | 8 (9,20%) |  |
| Imputation 4 | 2 or more RF | 60 (45,45%) | 52 (59,77%) | **0,037** |
|  | 1 RF | 52 (39,39%) | 27 (31,03%) |  |
|  | 0 RF | 20 (15,15%) | 8 (9,20%) |  |
| Imputation 5 | 2 or more RF | 62 (46,97%) | 52 (59,77%) | **0,047** |
|  | 1 RF | 49 (37,12%) | 27 (31,03%) |  |
|  | 0 RF | 21 (15,91%) | 8 (9,20%) |  |
| Imputation 6 | 2 or more RF | 60 (45,45%) | 52 (59,77%) | **0,022** |
|  | 1 RF | 51 (38,64%) | 28 (32,18%) |  |
|  | 0 RF | 21 (15,91%) | 7 (8,05%) |  |
| Imputation 7 | 2 or more RF | 60 (45,45%) | 52 (59,77%) | **0,037** |
|  | 1 RF | 52 (39,39%) | 27 (31,03%) |  |
|  | 0 RF | 20 (15,15%) | 8 (9,20%) |  |
| Imputation 8 | 2 or more RF | 62 (46,97%) | 52 (59,77%) | **0,047** |
|  | 1 RF | 49 (37,12%) | 27 (31,03%) |  |
|  | 0 RF | 21 (15,91%) | 8 (9,20%) |  |
| Imputation 9 | 2 or more RF | 60 (45,45%) | 52(59,77%) | **0,032** |
|  | 1 RF | 51 (38,64%) | 27(31,03%) |  |
|  | 0 RF | 21 (15,91%) | 8 (9,20%) |  |
| Imputation 10 | 2 or more RF | 61 (46,21%) | 52 (59,77%) | **0,039** |
|  | 1 RF | 50 (37,88%) | 27 (31,03%) |  |
|  | 0 RF | 21 (15,91%) | 8 (9,20%) |  |
| Imputation 11 | 2 or more RF | 60 (45,45%) | 52 (59,77%) | **0,032** |
|  | 1 RF | 51 (38,64%) | 27 (31,03%) |  |
|  | 0 RF | 21 (15,91%) | 8 (9,20%) |  |
| Imputation 12 | 2 or more RF | 60 (45,45%) | 52 (59,77%) | **0,022** |
|  | 1 RF | 51 (38,64%) | 28 (32,18%) |  |
|  | 0 RF | 21 (15,91%) | 7 (8,05%) |  |
| Imputation 13 | 2 or more RF | 62 (46,97%) | 52 (59,77%) | 0,054 |
|  | 1 RF | 50 (37,88%) | 27 (31,03%) |  |
|  | 0 RF | 20 (15,15%) | 8 (9,20%) |  |
| Imputation 14 | 2 or more RF | 60 (45,45%) | 52 (59,77%) | **0,032** |
|  | 1 RF | 51 (38,64%) | 27 (31,03%) |  |
|  | 0 RF | 21 (15,91%) | 8 (9,20%) |  |
| Imputation 15 * | 2 or more RF | 61 (46,21%) | 52 (59,77%) | **0,044** |
|  | 1 RF | 51 (38,64%) | 27 (31,03%) |  |
|  | 0 RF | 20 (15,15%) | 8 (9,20%) |  |
| Imputation 16 | 2 or more RF | 60 (45,45%) | 52 (59,77%) | **0,032** |
|  | 1 RF | 51 (38,64%) | 27 (31,03%) |  |
|  | 0 RF | 21 (15,91%) | 8 (9,20%) |  |
| Imputation 17 | 2 or more RF | 60 (45,45%) | 52 (59,77%) | **0,032** |
|  | 1 RF | 51 (38,64%) | 27 (31,03%) |  |
|  | 0 RF | 21 (15,91%) | 8 (9,20%) |  |
| Imputation 18 | 2 or more RF | 60 (45,45%) | 52 (59,77%) | **0,032** |
|  | 1 RF | 51 (38,64%) | 27 (31,03%) |  |
|  | 0 RF | 21 (15,91%) | 8 (9,20%) |  |
| Imputation 19 | 2 or more RF | 61(46,21%) | 53 (60,92%) | **0,029** |
|  | 1 RF | 50(37,88%) | 26 (29,89%) |  |
|  | 0 RF | 21(15,91%) | 8 (9,20%) |  |
| Imputation 20 | 2 or more RF | 61 (46,21%) | 52 (59,77%) | **0,044** |
|  | 1 RF | 51 (38,64%) | 27 (31,03%) |  |
|  | 0 RF | 20 (15,15%) | 8 (9,20%) |  |
| Imputation 21 | 2 or more RF | 61 (46,21%) | 52 (59,77%) | **0,028** |
|  | 1 RF | 50 (37,88%) | 28 (32,18%) |  |
|  | 0 RF | 21 (15,91%) | 7 (8,05%) |  |
| Imputation 22 | 2 or more RF | 60 (45,45%) | 52 (59,77%) | **0,032** |
|  | 1 RF | 51 (38,64%) | 27 (31,03%) |  |
|  | 0 RF | 21 (15,91%) | 8 (9,20%) |  |
| Imputation 23 | 2 or more RF | 61 (46,21%) | 52 (59,77%) | **0,044** |
|  | 1 RF | 51 (38,64%) | 27 (31,03%) |  |
|  | 0 RF | 20 (15,15%) | 8 (9,20%) |  |
| Imputation 24 | 2 or more RF | 60 (45,45%) | 52 (59,77%) | **0,032** |
|  | 1 RF | 51 (38,64%) | 27 (31,03%) |  |
|  | 0 RF | 21 (15,91%) | 8 (9,20%) |  |
| Imputation 25 | 2 or more RF | 61 (46,21%) | 52 (59,77%) | **0,039** |
|  | 1 RF | 50 (37,88%) | 27 (31,03%) |  |
|  | 0 RF | 21 (15,91%) | 8 (9,20%) |  |

* Represent imputation used for the main analysis in the paper

**Supplementary Table 2.** Multivariate Regression Analysis on 10-years CV Risk PC for each imputation

|  |  | β | 95% CL | | p-value |
| --- | --- | --- | --- | --- | --- |
| Imputation 1 | PR Cluster | 0,0677 | 0,0195 | 0,1158 | **0,006** |
|  | Risk Factors | 0,0178 | -0,0154 | 0,0511 | 0,292 |
| Imputation 2 | PR Cluster | 0,0677 | 0,0196 | 0,1158 | **0,006** |
|  | Risk Factors | 0,0183 | -0,0152 | 0,0518 | 0,284 |
| Imputation 3 | PR Cluster | 0,0670 | 0,0188 | 0,1151 | **0,007** |
|  | Risk Factors | 0,0205 | -0,0128 | 0,0538 | 0,226 |
| Imputation 4 | PR Cluster | 0,0676 | 0,0195 | 0,1158 | **0,006** |
|  | Risk Factors | 0,0182 | -0,0154 | 0,0517 | 0,288 |
| Imputation 5 | PR Cluster | 0,0683 | 0,0202 | 0,1165 | **0,006** |
|  | Risk Factors | 0,0151 | -0,0181 | 0,0484 | 0,370 |
| Imputation 6 | PR Cluster | 0,0668 | 0,0185 | 0,1150 | **0,007** |
|  | Risk Factors | 0,0204 | -0,0132 | 0,0540 | 0,233 |
| Imputation 7 | PR Cluster | 0,0667 | 0,0186 | 0,1148 | **0,007** |
|  | Risk Factors | 0,0227 | -0,0108 | 0,0562 | 0,184 |
| Imputation 8 | PR Cluster | 0,0679 | 0,0198 | 0,1160 | **0,006** |
|  | Risk Factors | 0,0173 | -0,0159 | 0,0505 | 0,306 |
| Imputation 9 | PR Cluster | 0,0670 | 0,0188 | 0,1151 | **0,007** |
|  | Risk Factors | 0,0205 | -0,0128 | 0,0538 | 0,226 |
| Imputation 10 | PR Cluster | 0,0669 | 0,0188 | 0,1150 | **0,007** |
|  | Risk Factors | 0,0218 | -0,0114 | 0,0550 | 0,197 |
| Imputation 11 | PR Cluster | 0,0670 | 0,0188 | 0,1151 | **0,007** |
|  | Risk Factors | 0,0205 | -0,0128 | 0,0538 | 0,226 |
| Imputation 12 | PR Cluster | 0,0668 | 0,0185 | 0,1150 | **0,007** |
|  | Risk Factors | 0,0204 | -0,0132 | 0,0540 | 0,233 |
| Imputation 13 | PR Cluster | 0,0668 | 0,0188 | 0,1148 | **0,007** |
|  | Risk Factors | 0,0241 | -0,0093 | 0,0575 | 0,156 |
| Imputation 14 | PR Cluster | 0,0670 | 0,0188 | 0,1151 | **0,007** |
|  | Risk Factors | 0,0205 | -0,0128 | 0,0538 | 0,226 |
| Imputation 15 * | PR Cluster | 0,0674 | 0,0193 | 0,1155 | **0,006** |
|  | Risk Factors | 0,0199 | -0,0136 | 0,0534 | 0,242 |
| Imputation 16 | PR Cluster | 0,0670 | 0,0188 | 0,1151 | **0,007** |
|  | Risk Factors | 0,0205 | -0,0128 | 0,0538 | 0,226 |
| Imputation 17 | PR Cluster | 0,0670 | 0,0188 | 0,1151 | **0,007** |
|  | Risk Factors | 0,0205 | -0,0128 | 0,0538 | 0,226 |
| Imputation 18 | PR Cluster | 0,0670 | 0,0188 | 0,1151 | **0,007** |
|  | Risk Factors | 0,0205 | -0,0128 | 0,0538 | 0,226 |
| Imputation 19 | PR Cluster | 0,0667 | 0,0186 | 0,1149 | **0,007** |
|  | Risk Factors | 0,0214 | -0,0119 | 0,0546 | 0,207 |
| Imputation 20 | PR Cluster | 0,0670 | 0,0190 | 0,1151 | **0,007** |
|  | Risk Factors | 0,0218 | -0,0117 | 0,0552 | 0,201 |
| Imputation 21 | PR Cluster | 0,0675 | 0,0193 | 0,1157 | **0,006** |
|  | Risk Factors | 0,0177 | -0,0159 | 0,0512 | 0,301 |
| Imputation 22 | PR Cluster | 0,0670 | 0,0188 | 0,1151 | **0,007** |
|  | Risk Factors | 0,0205 | -0,0128 | 0,0538 | 0,226 |
| Imputation 23 | PR Cluster | 0,0666 | 0,0186 | 0,1146 | **0,007** |
|  | Risk Factors | 0,0240 | -0,0094 | 0,0574 | 0,159 |
| Imputation 24 | PR Cluster | 0,0670 | 0,0188 | 0,1151 | **0,007** |
|  | Risk Factors | 0,0205 | -0,0128 | 0,0538 | 0,226 |
| Imputation 25 | PR Cluster | 0,0671 | 0,0190 | 0,1152 | **0,007** |
|  | Risk Factors | 0,0206 | -0,0126 | 0,0538 | 0,223 |

* Represent imputation used for the main analysis in the paper
